# Clinicians' preferences for managing aneurysmal subarachnoid hemorrhage using endothelin receptor antagonists

**DOI:** 10.3389/fneur.2023.1102290

**Published:** 2023-03-02

**Authors:** Sebastian Heidenreich, Myrto Trapali, Nicolas Krucien, Andrea Phillips-Beyer

**Affiliations:** ^1^Patient-Centered Research, Evidera, London, United Kingdom; ^2^Innovus Consulting, London, United Kingdom

**Keywords:** discrete choice experiment (DCE), aneurysmal subarachnoid hemorrhage, delayed cerebral ischemia, endothelin receptor antagonist (ERA), physician preference, clinician preference

## Abstract

**Background:**

The endothelin receptor antagonist (ERA) clazosentan is being investigated for the medical prevention of cerebral vasospasm and associated complications, such as delayed cerebral ischemia (DCI), after aneurysmal subarachnoid hemorrhage (aSAH). This study quantified how clinicians weigh the benefits and risks of ERAs for DCI prevention to better understand their treatment needs and expectations.

**Methods:**

An online choice experiment was conducted to elicit preferences of neurologists, intensivists, and neurosurgeons treating aSAH in the US and UK for the use of ERAs. The design of the choice experiment was informed by a feasibility assessment (*N* = 100), one-on-one interviews with clinicians (*N* = 10), a qualitative pilot (*N* = 13), and a quantitative pilot (*N* = 50). Selected treatment attributes included in the choice experiment were: one benefit (likelihood of DCI); and three risks (lung complications, hypotension, and anemia). In the choice experiment, clinicians repeatedly chose best and worst treatment options based on a scenario of a patient being treated in the ICU after aneurism repair. A correlated mixed logit model determined the relative attribute importance (RAI) and associated highest density interval (HDI) as well as acceptable benefit-risk trade-offs.

**Results:**

The final choice experiment was completed by 350 clinicians (116 neurologists, 129 intensivists/intensive care clinicians, and 105 neurosurgeons; mean age, 47.4 years). Reducing the likelihood of DCI (RAI = 56.5% [HDI, 53.6–59.5%]) had the largest impact on clinicians' treatment choices, followed by avoiding the risks of lung complications (RAI = 29.6% [HDI, 27.1–32.3%]), hypotension (RAI = 9.2% [HDI, 7.5–10.8%]), and anemia (RAI = 4.7% [HDI, 3.7–5.8%]). Clinicians expected the likelihood of DCI to decrease by ≥8.1% for a 20% increase in the risk of lung complications, ≥2.4% for a 20% increase in the risk of hypotension, and ≥1.2% for a 20% increase in the risk of anemia.

**Conclusions:**

Clinicians were willing to accept certain increased risks of adverse events for a reduced risk of DCI after aSAH. The likelihood of DCI occurring after aSAH can therefore be considered a clinically relevant endpoint in aSAH treatment development. Thus, evaluations of ERAs might focus on whether improvements (i.e., reductions) in the likelihood of DCI justify the risks of adverse events.

## Introduction

Cerebral vasospasm is a common complication following aneurysmal subarachnoid hemorrhage (aSAH), occurring in up to 70% of patients (1). In 20% to 50% of patients with aSAH, cerebral vasospasm leads to delayed cerebral ischemia (DCI), which may progress to cerebral infarction, leading to motor deficits, cognitive dysfunction, other complications, and death. DCI is the most common preventable cause of death and poor neurological outcome in patients who survive aSAH (1).

The current standard of care in the US and EU for preventing DCI after aSAH is oral nimodipine, a calcium channel blocker, in conjunction with maintenance of euvolemia (2, 3). Nimodipine has been used since the late 1980s to prevent secondary ischemic complications of aSAH and, although most guidelines agree that it improves outcomes in patients, its mechanism of action is unknown, and it does not prevent or treat cerebral vasospasm (4–6). Endovascular therapy has also been shown to provide immediate improvements in DCI, but the effect is often not durable, and complications such as thrombosis or vessel rupture raise concern about the benefit-risk balance (7, 8).

Based on the role of endothelin-1 and its type A receptor in the pathogenesis of aSAH-induced cerebral vasospasm, the endothelin receptor antagonist (ERA) clazosentan is being investigated for the prevention of DCI after aSAH (9–11). For example, high doses of clazosentan have been shown to reduce the incidence of the vasospasm-related delayed ischemic neurological deficit and new cerebral infarction. Clazosentan was approved in Japan in early 2022 for the prevention of cerebral vasospasm, vasospasm-related cerebral infarction, and cerebral ischemic symptoms after aSAH (12). Additional data on the efficacy and safety of clazosentan is being collected in the ongoing REACT trial (NCT03585270) (13). Although ERAs are potentially beneficial in preventing vasospasm-related morbidity after aSAH, their use is associated with some adverse events (11, 14, 15). General adverse events of ERAs are related to their vasodilator properties and fluid retention and include flushing, nausea, headache, nasal congestion, and peripheral edema, as well as hypotension and palpitations (10). Other adverse events include lung complications (e.g., pleural effusion, pulmonary edema, and pneumonia), anemia due to plasma volume expansion, and systemic vasodilatory-induced hypotension (10, 11). With ERAs being a new treatment class within the context of aSAH, little is known about the relative importance that clinicians place on these adverse events compared to efficacy.

Preference studies are increasingly used to facilitate the interpretation of clinical data by determining if treatment benefits outweigh total treatment risks from the perspective of different stakeholders (16, 17). Such quantitative preference information is often obtained using choice experiments, in which participants are presented with a series of tasks in which they choose between hypothetical treatment options that require trade-offs between risks and benefits. This study explored the preferences of clinicians involved in the management of aSAH post aneurysm repair, the benefit-risk of ERAs from the clinicians' perspective, and the trade-offs clinicians are willing to make in risks of adverse events to obtain a reduction in the risk of DCI. The results provide insights into treatment needs and expectations from the clinicians' perspective. Acceptable benefit-risk trade-offs obtained in this study can be used in future quantitative benefit-risk assessment to help understand whether clinicians consider observed levels of ERA efficacy as sufficient to outweigh observed adverse events.

## Methods

### Overview

An online choice experiment was conducted between November 26, 2020 and February 17, 2021 to elicit the preferences of clinicians in the US and UK for the treatment of DCI using ERAs and the benefit-risk trade-offs they are willing to make. To be eligible for study participation, clinicians had to be a licensed and actively practicing neurologist, intensivist, or neurosurgeon in the US or UK, had to report experience treating at least two patients with aSAH in the past 10 years, had to report experience with treating at least one aSAH patient within 14 days of the diagnosis in the past 10 years, and had to report being familiar with interpreting DCI. All participants were recruited via commercially managed physician access panels. A random sample of neurologists, neurosurgeons, and intensivists registered with the access panel were invited by email to complete an online screening questionnaire and an informed consent form if they met the eligibility criteria. The recruitment strategy was tested at the beginning of the study in a feasibility survey among 100 clinicians, which also provided preliminary insights into the relative importance of different treatment attributes (see Supplementary material).

The main choice experiment was developed and tested in a multi-phased approach. First, qualitative interviews with 10 clinicians were conducted to inform the development of a patient scenario used for the main preference elicitation. A qualitative pilot was subsequently conducted with 13 clinicians to explore the clarity and relevance of the patient scenario and definitions and the wording of benefits and risks included in the choice experiment, along with clinicians' willingness to make trade-offs. Adjustments were made to the choice experiment based on clinicians' input. The survey was fielded among 50 clinicians as a quantitative pilot and this same version was used in the main study of 300 clinicians.

### Choice experiment

Benefits and risks of ERA treatment in aSAH were characterized as treatment attributes and included in the choice experiment at several levels (e.g., 10% or 20% risk of anemia) (Table 1). The selection of these treatment attributes was informed by one-on-one qualitative interviews conducted in May 2020 with 10 clinicians. The interviews consisted of three parts. Part 1 discussed clinicians' experience with treating aSAH. Part 2 focused specifically on the initial management as well as the management of cerebral vasospasm. Part 3 included a hypothetical choice task in which clinicians made tradeoffs between the likelihood of DCI and the risks of lung complications, hypotension, and anemia. All interviews were audio recorded, transcribed verbatim, and analyzed using a thematic approach. The analysis of the qualitative interviews confirmed the relevance and completeness of the identified attributes. More details on the qualitative interviews are included in the Supplementary material.

**Table 1:** Attributes and levels in the choice experiment.

**Attribute**	**Description**	**Levels**
Likelihood of DCI	The efficacy of an ERA can be measured by its ability to prevent the occurrence of clinical deterioration due to DCI from cerebral vasospasms in patients with aSAH.	12 out of 100 (12%)
		15 out of 100 (15%)
		18 out of 100 (18%)
		21 out of 100 (21%)
		24 out of 100 (24%)
		27 out of 100 (27%)
		30 out of 100 (30%)
Risk of hypotension	Some patients develop mild to moderate hypotension in the order of 10% reduction in blood pressure that can be corrected by vasopressor and fluid therapy. Patients rarely discontinue ERAs due to hypotension.	2 out of 100 (2%)
		8 out of 100 (8%)
		16 out of 100 (16%)
		24 out of 100 (24%)
Risk of anemia	Anemia is a class effect of ERA and is attributed to plasma volume expansion as a results of fluid retention. It is typically reversible after discontinuation of ERA and does not require blood transfusion.	10 out of 100 (10%)
		20 out of 100 (20%)
		30 out of 100 (30%)
		40 out of 100 (40%)
Risk of lung complications	Patients may develop lung complications such as pleural effusions, pulmonary edema, and pneumonia. Euvolemia can be used for the management of these lung complications within a typical ICU setting.	20 out of 100 (20%)
		30 out of 100 (30%)
		40 out of 100 (40%)
		50 out of 100 (50%)

aSAH, aneurysmal subarachnoid hemorrhage; DCI, delayed cerebral ischemia; ERA, endothelin-1 receptor antagonist; ICU, intensive care unit.

**Figure 1:**
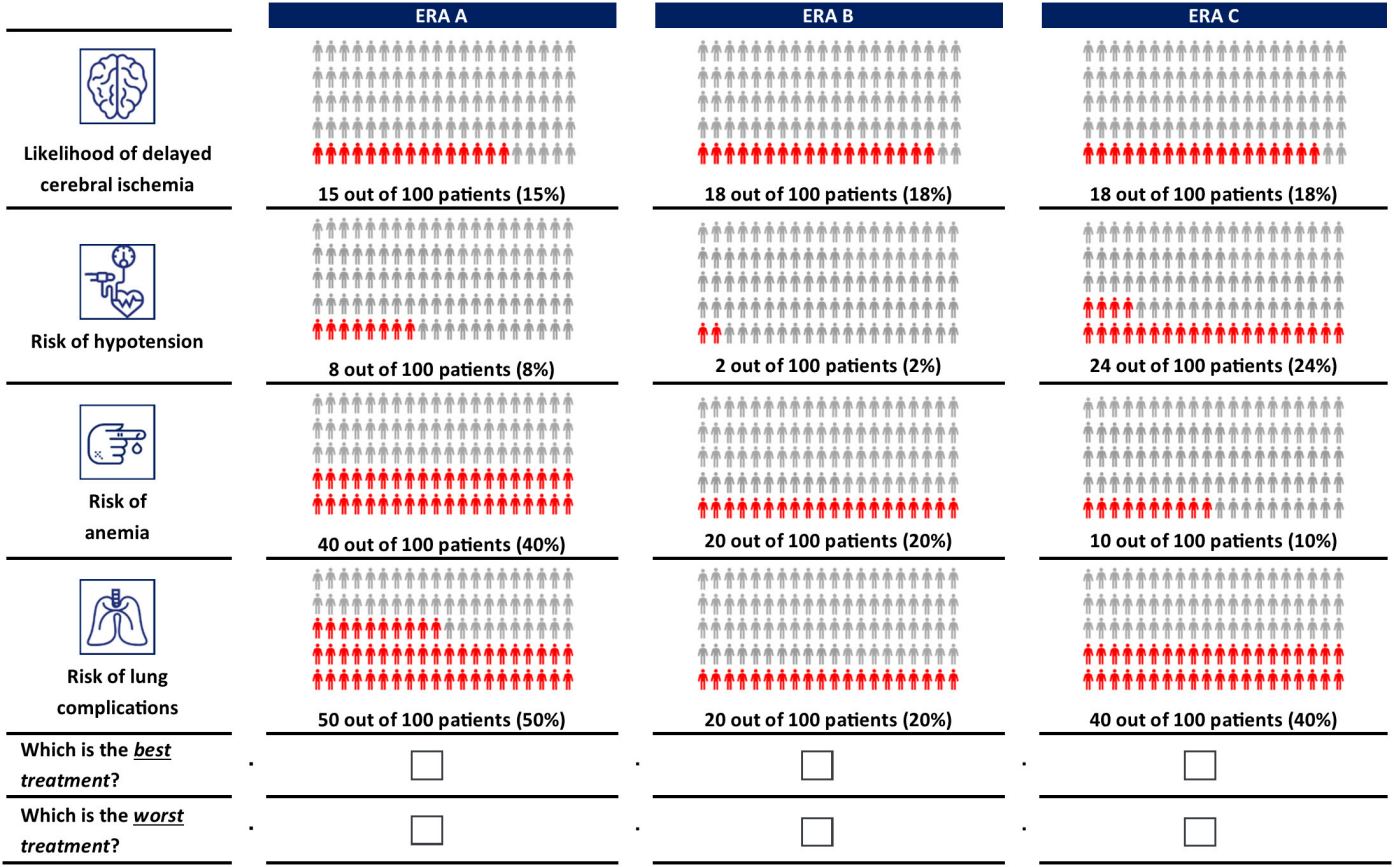
Example choice task.

A D-efficient design (18) was generated in Ngene version 1.2.1 (ChoiceMetrics, Sydney, Australia) to combine the treatment attributes and levels into 14 choice tasks. In each choice task, clinicians were asked to choose the best and worst of three hypothetical ERA options. Each ERA option was described by different levels of each attribute. The order of the experimental choice tasks, the ERA options, and attributes was randomized among clinicians to avoid ordering effects (19, 20). Clinicians completed a practice choice task before advancing to the main choice experiment. An example choice task is shown in Figure 1. More details on the experimental design are included in the Supplementary material.

### Online choice experiment survey

In the online choice experiment survey, participants were first informed about the purpose of the study and the benefits and risks of ERAs. Before completing the actual choice experiment, all clinicians were presented with the following patient scenario:

*Patient J.J. is a 50-year-old man with a past medical history of hypertension. On arrival in the emergency department, he complained of the “worst headache of his life”. He appeared confused but arousable and had no other evidence of neurological deficits. His initial Hunt and Hess grade was II, and his Glasgow Coma Scale score was 13. An admission computed tomography scan of the brain revealed acute subarachnoid hemorrhage due to leakage from a 5-mm anterior artery aneurysm (Fisher grade I). J.J. was transferred to the intensive care unit, where he underwent coiling of his aneurysm. There were no complications during the surgery*.

Additional questionnaires collected sociodemographic data, clinical experience, and the expected effect of reducing the risk of DCI after an aSAH event. Questions included “How do you perceive the need for a new pharmacological treatment in the routine care of aSAH patients?” on a scale of 0 (not needed) to 10 (very much needed); the number of days in response to “I think that by avoiding DCI and its associated complications, treatment with an ERA can potentially reduce the ICU stay of patients”; and the number of days in response to “I think that by avoiding DCI and its associated complications, treatment with an ERA can potentially reduce the overall hospital stay of patients”. The full choice experiment survey is included as Supplementary material.

### Qualitative and quantitative pre-testing

The survey was qualitatively pre-tested in 60-min computer-assisted interviews with 13 clinicians (six in the UK, seven in the US). Clinicians were asked to “think aloud” while completing the survey online and sharing their screens with interviewers. Using a semi-structured interview guide, interviewers also probed clinicians on the clarity of survey instructions; their understanding of the survey content, questions and choice tasks; the completeness of response options; and the relevance of each attribute included in the preference elicitation tasks. Clinicians were asked if they perceived the choice tasks to be complete or whether any relevant information or concepts were missing from their perspective. The qualitative interviews were used to iteratively adjust the format of the different choice questions. Clinicians were initially presented with the choice between two ERAs and an opt-out (i.e., no ERA) alternative. Based on clinicians' input, the opt-out option was removed and replaced by a third ERA because it was never preferred by any clinician. Due to the high importance that clinicians placed on the likelihood of DCI, the maximum difference in the levels across alternatives was constrained at 3% to facilitate the identification of benefit-risk trade-offs with attributes of lower relative importance. Furthermore, the likelihood of DCI was constrained to overlap between two alternatives to ensure that the relative importance of the different adverse events risk could be identified. All updates were made iteratively and tested in subsequent interviews. The survey was subsequently piloted with 50 respondents to test the appropriateness of selected attribute levels. No additional changes were made after the quantitative pilot. More details on the pre-testing are included in the Supplementary material.

### Statistical analysis

Choice experiment data from the quantitative pilot (*N* = 50) were merged with data from the main study (*N* = 300) for analysis, yielding choice experiment data from a total of 350 clinicians. Data from the choice experiment were analyzed based on random utility maximization theory, wherein preferences were represented as a linear function of the treatment attributes and an extreme-value type I distributed error (21). The effects of changes in benefits and risks on the treatment utility were estimated using a sequential Bayesian mixed logit model with a multivariate log-normal distribution to account for heterogeneity in preferences and choice consistency (22, 23). Median relative attribute importance (RAI) scores were computed for each attribute to assess their maximum contribution to treatment utility. The scores were normalized to sum to 100%, and highest density intervals (HDIs) were obtained from the posterior distribution. The model was rearranged into valuation space to directly estimate the median minimum acceptable reduction in the likelihood of DCI for increases in the risks of lung complications, hypotension, and anemia (10, 11, 24).

Subgroup analysis was conducted to explore if preferences differed by country (US/UK) and clinicians' specialty (neurologists/intensivists/neurosurgeons). This was achieved by interacting clinician's characteristics with the attributes in the model and conducting a likelihood-ratio test.

All models were estimated with R software (version 4.0.2) using a Monte Carlo Markov Chain simulation procedure for hierarchical Bayesian logit from the Apollo 0.2.6 package (25). The adjusted McFadden pseudo R^2^ was used to assess goodness of fit. Further details of the statistical analysis are provided in the Supplementary material.

## Results

### Sample characteristics

**Figure 2:**
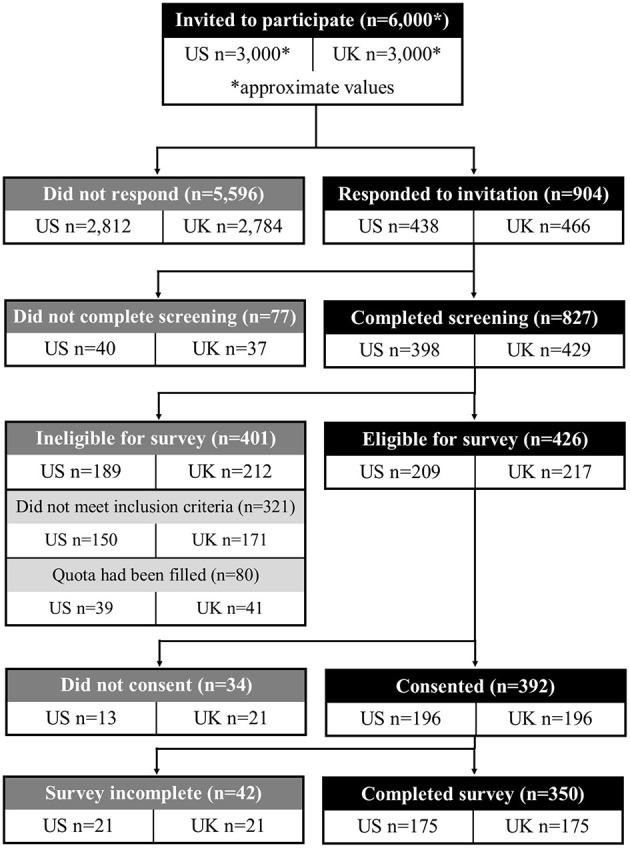
Participant disposition diagram. Participant recruitment and inclusion for the main survey.

The survey was completed by 116 neurologists, 129 intensivists/intensive care clinicians, and 105 neurosurgeons who were recruited via commercially managed access panels (Table 2). The recruitment, screening, and survey completion process is summarized in Figure 2. The mean age was 47.4 ± 8.5 years. Most clinicians had been in practice for more than 10 years (86%), had seen more than 20 aSAH patients over the past 10 years (82%), and had seen more than 20 aSAH patients within 14 days of diagnosis over the past 10 years (71%).

**Table 2:** Sample characteristics.

	**Overall sample**	**UK**	**US**
**Characteristic**	**(*****N** =* **350)**	**(*****N** =* **175)**	**(*****N** =* **175)**
Mean age (standard deviation)	47.4 (8.5)	46.0 (7.6)	48.8 (9.1)
Time in practice, *n* (%)			
< 5 y	4 (1.1)	2 (1.1)	2 (1.1)
5–10 y	44 (12.6)	14 (8.0)	30 (17.1)
10–20 y	181 (51.7)	102 (58.3)	79 (45.1)
>20 y	121 (34.6)	57 (32.6)	64 (36.6)
Area of specialty, *n* (%)			
Neurologist	116 (33.1)	63 (36.0)	53 (30.3)
Intensivist/intensivecare clinician	129 (36.9)	67 (38.3)	62 (35.4)
Neurosurgeon	105 (30.0)	45 (25.7)	60 (34.3)
Number of aSAH patients seen in the past 10 y, *n* (%)			
2–5	9 (2.6)	5 (2.9)	4 (2.3)
6–10	15 (4.3)	7 (4.0)	8 (4.6)
11–15	21 (6.0)	7 (4.0)	14 (8.0)
16–20	17 (4.9)	9 (5.1)	8 (4.6)
>20	288 (82.3)	147 (84.0)	141 (80.6)
Number of aSAH patients within 14 days of diagnosis in the past 10 y, *n* (%)			
1–5	36 (10.3)	21 (12.0)	15 (8.6)
6–10	23 (6.6)	7 (4.0)	16 (9.1)
11–15	21 (6.0)	7 (4.0)	14 (8.0)
16–20	22 (6.3)	12 (6.9)	10 (5.7)
>20	248 (70.9)	128 (73.1)	120 (68.6)

aSAH, aneurysmal subarachnoid hemorrhage.

### Clinicians' expectations for pharmacological treatment of DCI

**Figure 3:**
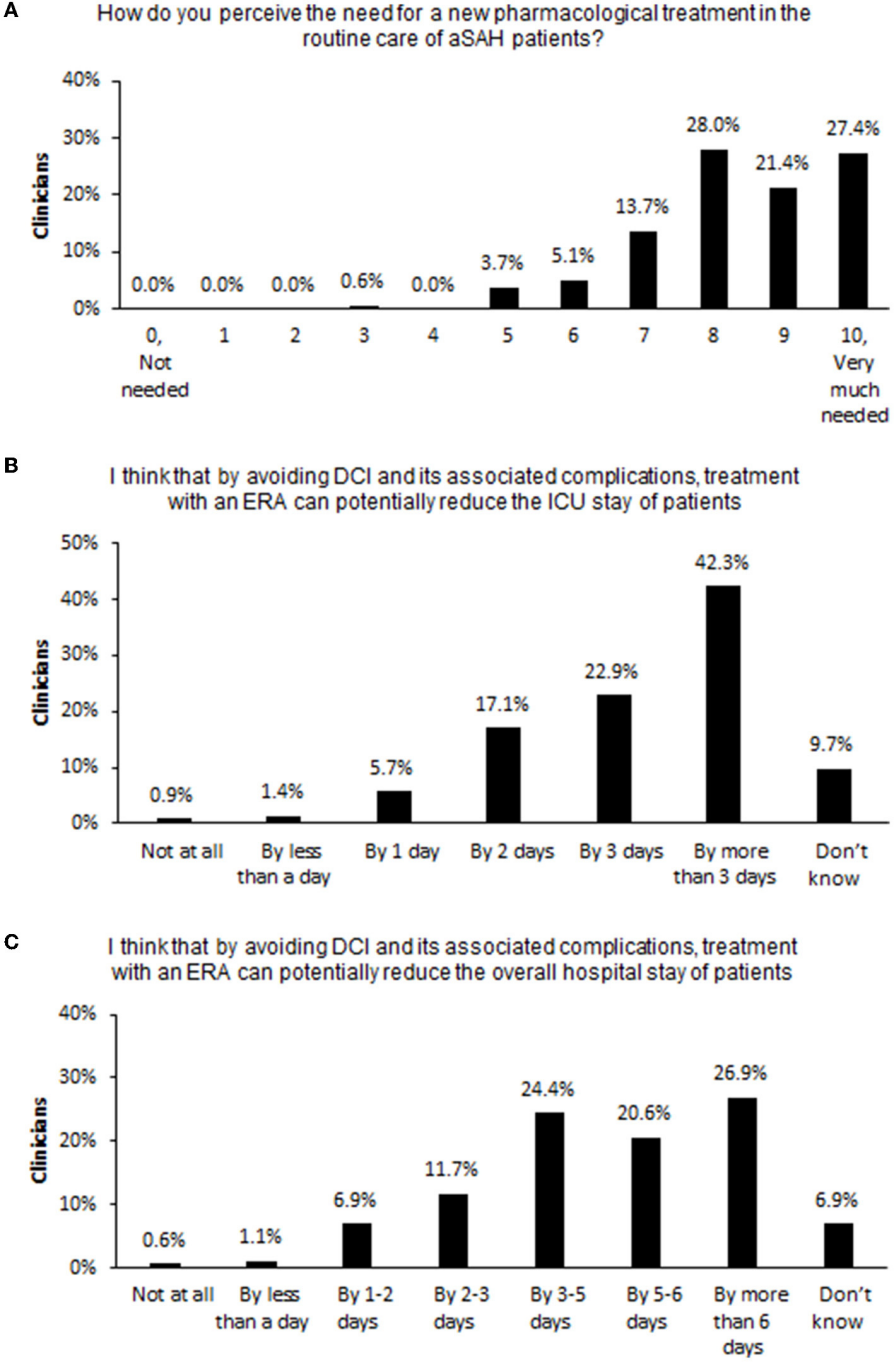
Clinicians' expectations of pharmacological treatments for DCI. Percent of surveyed clinicians endorsing each choice option in response to questions **(A)** on the need for a new pharmacological treatment for aSAH, **(B)** on the potential reduction of patients' length of ICU stay if DCI can be prevented, and **(C)** on the potential reduction of patients' overall hospital stay if DCI can be prevented. aSAH, aneurysmal subarachnoid hemorrhage; DCI, delayed cerebral ischemia; ERA, endothelin-1 receptor antagonist; ICU, intensive care unit.

On a scale of 0 (not needed) to 10 (very much needed), nearly half of clinicians (49%) rated the need for a new pharmacological treatment as 9 or 10, and 91% rated the need as 7 or more (Figure 3A). Most clinicians thought that by avoiding DCI and associated complications, treatment with an ERA could potentially reduce patients' stay in the intensive care unit (ICU) by 3 or more days (65%; Figure 3B) and reduce patients' overall hospital stay by at least 3–5 days (73%; Figure 3C).

### Treatment preferences

**Figure 4:**
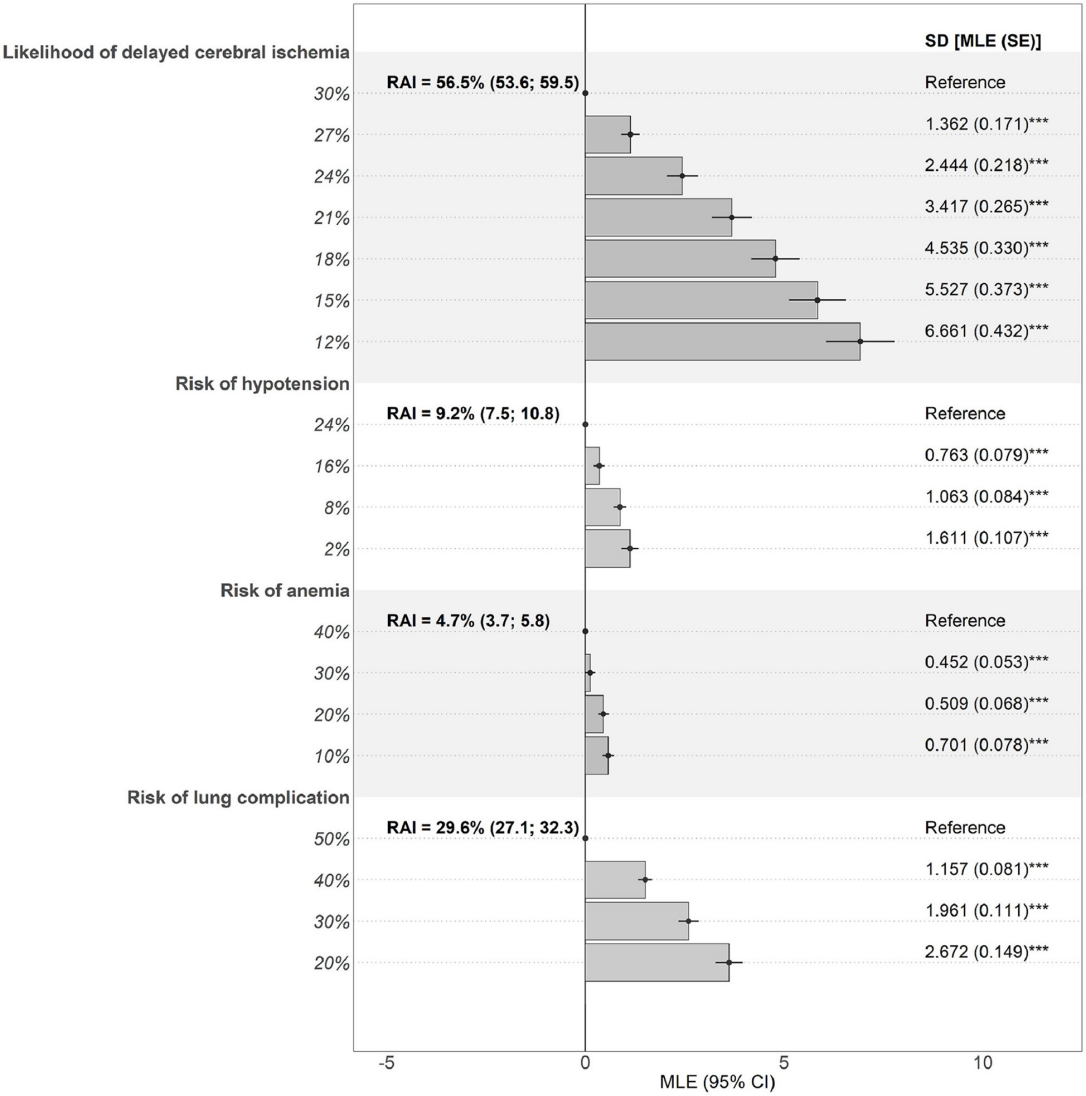
Preferences and RAIs for benefit and risk attributes. Estimates are the effect of deviations from a reference level (dot) on preferences. Bars denote mean effects. Significant SDs denote presence of preference heterogeneity. CI, confidence interval; MLE, maximum likelihood estimate; RAI, relative attribute importance; SD, standard deviation; SE, standard error. ***p < 0.001.

The data fit for the main model was good (adjusted McFadden R^2^ = 0.484; Supplementary Table S1), suggesting it was able to explain the preferences that clinicians denoted in the choice experiment. Estimated effects were significant for all attributes (*p* < 0.001), implying that they all jointly influenced treatment choices (Figure 4).

Reducing the likelihood of DCI (RAI = 56.5% [HDI, 53.6–59.5%]) had the largest impact on clinicians' treatment choices, followed by avoiding the risks of lung complications (RAI = 29.6% [HDI, 27.1–32.3%]), hypotension (RAI = 9.2% [HDI, 7.5–10.8%]), and anemia (RAI = 4.7% [HDI, 3.7–5.8%]) (Figure 4 and Supplementary Table S2). Overall, the possibility of reducing the likelihood of DCI had, on average, a larger impact on the choice of treatment than all the risks together (combined RAI = 43.5% [HDI, 40.5–46.4%]; *p* < 0.001). Among the treatment risks, lung complications had a 2.1-fold (HDI, 1.8–2.6; *p* < 0.001) greater effect on treatment preferences than hypotension and anemia combined (joint RAI = 13.9% [HDI, 11.9–15.9%]; *p* < 0.001). However, standard deviations for estimated parameters were significant, indicating that preferences were clinician specific.

Preferences were not affected by clinician specialty but were affected by country of origin (Supplementary Table S3). Clinicians from the US placed more importance on reduction in DCI (RAI = 62.2% vs. 48.9%; *p* < 0.05) but less importance on the risk of lung complications (RAI = 24.1 vs. 37.0%; *p* < 0.01) than clinicians from the UK.

### Benefit-risk trade-offs

For a 5% increase in the risk of lung complications, clinicians would expect the likelihood of DCI to decrease by at least 2.0%, and for a 20% increase in the risk of lung complications, they would expect it to decrease by at least 8.1% (Table 3).

**Table 3:** Minimum acceptable reduction of DCI for increases in risks of adverse events.

**Risk increase (%)**	**Minimum acceptable reduction in the likelihood of DCI: % [95% confidence interval]**		
	**Risk of hypotension**	**Risk of anemia**	**Risk of lung complications**
1	0.12 [0.09; 0.15]	0.06 [0.04; 0.08]	0.40 [0.33; 0.48]
5	0.60 [0.45; 0.75]	0.31 [0.22; 0.39]	2.02 [1.66; 2.38]
10	1.21 [0.90; 1.51]	0.61 [0.44; 0.79]	4.05 [3.33; 4.77]
15	1.81 [1.35; 2.26]	0.92 [0.65; 1.18]	6.07 [4.99; 7.15]
20	2.41 [1.80; 3.02]	1.23 [0.87; 1.58]	8.10 [6.66; 9.54]
25	3.01 [2.25; 3.77]	1.53 [1.09; 1.97]	10.12 [8.32; 11.92]
30	3.62 [2.71; 4.53]	1.84 [1.31; 2.37]	12.15 [9.99; 14.30]
35	4.22 [3.16; 5.28]	2.14 [1.53; 2.76]	14.17 [11.65; 16.69]
40	4.82 [3.61; 6.04]	2.45 [1.74; 3.16]	16.19 [13.32; 19.07]

Log likelihood = −5,224.5; Bayesian information criterion = 10,596; adjusted McFadden pseudo R^2^ = 51.33%; p < 0.001 for all analyses.

DCI, delayed cognitive impairment.

For increased risks of hypotension or anemia, the required decreases in the likelihood of DCI were lower. Specifically, for a 5% increase in the risk of hypotension, clinicians would expect the likelihood of DCI to decrease by 0.6%, and for a 20% increase in risk of hypotension, they would expect the likelihood of DCI to decrease by at least 2.4%. For a 5% increase in the risk of anemia, clinicians would expect the likelihood of DCI to decrease by 0.3%, and for a 20% increase in the risk of anemia, they would expect the likelihood of DCI to decrease by 1.2%. Finally, for a treatment expected to increase the risks of lung complications by 20%, hypotension by 20%, and anemia by 40%, clinicians would expect the likelihood of DCI to decrease by at least 13.0%.

## Discussion

Based on the role of endothelin and its receptor in the pathogenesis of aSAH-induced cerebral vasospasm, the ERA clazosentan is being investigated for the prevention of DCI after aSAH. (9) This quantitative study shows that clinicians treating aSAH value the potential benefit of ERAs in reducing the likelihood of DCI and accept certain risks of lung complications, hypotension, and anemia. However, acceptable trade-offs varied among clinicians and were individual specific. Further, clinicians from the US valued the likelihood of a reduction in DCI more than clinicians from the UK. Despite the heterogeneity in preferences, these findings emphasize the importance of DCI as an outcome to clinicians. Notably, the patient scenario used in the study assumed a Fisher grade of I and a Glasgow Coma Scale score of 13. More severe scenarios are expected to result in DCI having even more importance than the treatment risks considered in this study. On the other hand, the scenario is concerned with an ICU setting and generalization to a lower ward or home setting may not be applicable.

The study also estimated the minimum acceptable efficacy of a treatment in reducing the likelihood of DCI. These data can help formulate preference-based product profiles, be used as a basis for quantitative patient-centered benefit-risk assessments, and help develop decision aids to support routine management of aSAH.

Nimodipine is often considered for the prevention of secondary ischemic complications of aSAH and, although it may improve outcomes, its mechanism of action is unknown, and it does not prevent or treat cerebral vasospasm (4–6). Endovascular therapy has been shown to provide immediate improvements in DCI, but the effect is frequently not durable, and its use is associated with serious complications, such as thrombosis and vessel rupture (7, 8). The current study suggests that, although ERAs have potential adverse effects, clinicians consider that their benefits would outweigh their risks.

The study design followed best practice in quantitative preference elicitation by taking a multistage approach in which clinician input was considered in the study design through concept elicitation interviews and pre-testing. The study benefitted from including a sample of neurologists, neurosurgeons, and intensivists who were experienced in treating aSAH patients and familiar with cerebral vasospasm and DCI. As part of the study, clinicians were presented with a patient scenario, which was developed based on clinician input and served as a reference point for applying the findings.

A limitation of this study is that selection bias cannot be completely ruled out, because the preferences of clinicians who decided not to participate could have been different from clinicians who did participate. Further, no test of internal choice consistency was conducted in this study, which means that no insights into preference stability or preferential learning can be generated. Finally, an opt-out alternative had to be removed from the choice experiment after the cognitive pilot due to not being considered as realistic or desirable by clinicians. This underlines the perceived need for medical treatments for the management of aSAH.

In conclusion, this study showed that clinicians expect an effective treatment to prevent DCI and reduce inpatient stays in the ICU or hospital, which is supported by previous clinical research (26). The ability of treatments to prevent DCI appears to be the primary driver of how clinicians value such treatments, while the risk of lung complications is an important safety concern. As the first study to examine clinicians' willingness to trade off benefits and risks in managing aSAH post aneurism repair, it makes an important contribution to establishing future treatment priorities.

## Data availability statement

The raw data supporting the conclusions of this article will be made available by the authors, without undue reservation.

## Ethics statement

Ethical review and approval was not required for the study on human participants in accordance with the local legislation and institutional requirements. The patients/participants provided their written informed consent to participate in this study.

## Author contributions

SH and AP-B conceived of and designed the study. SH, MT, and AP-B implemented the study. MT acquired the data. NK performed quantitative analysis on the data. All authors contributed to interpreting the data.
