# Supplementary material for: Clinicians' preferences for managing aneurysmal subarachnoid hemorrhage using endothelin receptor antagonists
Source: Front Neurol. 2023 Mar 2;14:1102290. doi: 10.3389/fneur.2023.1102290 (PMC10017541; doi:10.3389/fneur.2023.1102290)
Supplement: Supplementary file 1 [file Data_Sheet_1.pdf]

Supplemental Tables and Figures

Table S1. Main Model Estimates

		**Estimate (SE)**		**SD of underlying distribution**	
Attribute	*Level*	*Mean (SE)*	*95% CI*	*Mean (SE)*	*95% CI*
Alternative specific constant	ERA A	-0.015 (0.049)	-0.111; 0.080	-	-
	ERA B	0.105 (0.040)**	0.026; 0.183	-	-
	ERA C	Reference	-	-	-
Risk of anemia	10%	0.574 (0.075)***	0.427; 0.720	0.701 (0.078)***	0.548; 0.854
	20%	0.451 (0.069)***	0.316; 0.586	0.509 (0.068)***	0.376; 0.641
	30%	0.120 (0.067)	-0.011; 0.252	0.452 (0.053)***	0.348; 0.557
	40%	Reference	-	-	-
Likelihood of DCI	12%	6.916 (0.437)***	6.061; 7.772	6.661 (0.432)***	5.814; 7.508
	15%	5.839 (0.367)***	5.120; 6.557	5.527 (0.373)***	4.795; 6.259
	18%	4.782 (0.312)***	4.170; 5.394	4.535 (0.330)***	3.888; 5.182
	21%	3.683 (0.256)***	3.180; 4.185	3.417 (0.265)***	2.897; 3.936
	24%	2.439 (0.199)***	2.048; 2.829	2.444 (0.218)***	2.016; 2.872
	27%	1.133 (0.122)***	0.895; 1.371	1.362 (0.171)***	1.026; 1.698
	30%	Reference	-	-	-
Risk of lung complications	20%	3.616 (0.173)***	3.277; 3.954	2.672 (0.149)***	2.379; 2.964
	30%	2.594 (0.131)***	2.338; 2.850	1.961 (0.111)***	1.744; 2.178
	40%	1.503 (0.089)***	1.329; 1.678	1.157 (0.081)***	0.999; 1.316
	50%	Reference	-	-	-
Risk of hypotension	2%	1.120 (0.112)***	0.900; 1.339	1.611 (0.107)***	1.402; 1.821
	8%	0.867 (0.081)***	0.708; 1.025	1.063 (0.084)***	0.898; 1.229
	16%	0.347 (0.071)***	0.207; 0.487	0.763 (0.079)***	0.608; 0.918
	24%	Reference	-	-	-

Log likelihood = -4397.7; adjusted McFadden pseudo R^2 = 0.484; Bayesian information criteria = 10054.5
Abbreviations: DCI, delayed cerebral ischemia; ERA, endothelin receptor antagonist; SD, standard deviation; SE, standard error
*** p <0.001, ** p <0.01, * p <0.05%

Table S2. Minimum Acceptable Reduction in Likelihood of DCI

		Risk of anemia		Likelihood of DCI		Risk of hypotension		Risk of lung complications	
**Characteristic**	**Level**	**RAI (SE)**	**dRAI (SE)**	**RAI (SE)**	**dRAI (SE)**	**RAI (SE)**	**dRAI (SE)**	**RAI (SE)**	**dRAI (SE)**
Country of residence	US	3.92 (0.68)	-0.58 (0.88)	62.24 (1.83)	5.74* (2.38)	9.76 (0.96)	0.45 (1.24)	24.08 (1.47)	-5.62** (1.97)
	UK	5.41 (0.80)	0.92 (0.98)	48.91 (2.66)	-7.60* (3.06)	8.71 (1.20)	-0.59 (1.43)	36.97 (2.29)	7.27** (2.64)
Medical specialty	Neurologist	5.27 (0.83)	0.63 (0.99)	54.86 (2.64)	-1.55 (3.06)	8.72 (1.39)	-0.59 (1.60)	31.15 (2.52)	1.51 (2.87)
	Intensivist	4.15 (0.71)	-0.50 (0.89)	58.20 (2.30)	1.79 (2.76)	9.55 (1.50)	0.24 (1.69)	28.10 (2.41)	-1.54 (2.78)
	Neurosurgeon	5.01 (0.91)	0.37 (1.05)	55.88 (2.07)	-0.54 (2.58)	9.51 (1.42)	0.21 (1.63)	29.60 (2.03)	-0.04 (2.46)

Log likelihood = -4397.7; adjusted McFadden pseudo R^2 = 48.35; Bayesian information criteria = 10054.5

Abbreviations: dRAI, RAI difference; RAI: relative attribute importance; SE, standard error
*** p <0.001, ** p <0.01, * p <0.05%

Table S3. Subgroup Analyses

Attribute	Level	Estimate (SE) Overall Mean	Overall SD	Interaction (difference to overall estimate) Intensivist	Neurosurgeon	Neurologist	Interaction (difference to overall estimate) UK	US
Constant	ERA A	-0.008 (0.027)	-	-	-	-	-	-
	ERA B	0.096*** (0.019)	-	-	-	-	-	-
	ERA C	Reference	-	-	-	-	-	-
Likelihood of DCI	1% reduction	1.174*** (0.243)	5.934* (2.516)	-0.022** (0.007)	-0.006 (0.010)	0.028** (0.011)	0.016 (0.009)	-0.016 (0.009)
Risk of lung complications	1% reduction	0.154*** (0.013)	0.203*** (0.034)	0.005 (0.003)	-0.005 (0.004)	0.000 (0.005)	0.006 (0.004)	-0.006 (0.004)
Risk of hypotension	1% reduction	0.097*** (0.017)	0.342* (0.135)	0.001 (0.003)	0.001 (0.004)	-0.002 (0.005)	0.002 (0.004)	-0.002 (0.004)
Risk of anemia	1% reduction	0.038*** (0.007)	0.103* (0.042)	0.002 (0.002)	-0.001 (0.002)	0.000 (0.003)	-0.001 (0.003)	0.001 (0.003)

Log likelihood = 4700; adjusted McFadden pseudo R^2 = 0.462; Bayesian information criterion = 9657.4
Abbreviations: DCI, delayed cerebral ischemia; ERA, endothelin receptor antagonist; SD, standard deviation; SE, standard error
*** p <0.001, **p <0.01, *p <0.05

Supplemental Methods

Qualitative Interviews

Ten one-on-one qualitative interviews with clinicians were conducted in May 2020 to test if the key outcomes from CONCIOUS-1 and CONCIOUS-2 trials presented were clinician-relevant and provided enough information for making treatment decisions. The qualitative interviews also aimed to provide rich qualitative data that could be used to contextualize the quantitative preference findings. Furthermore, clinicians' maximum acceptable risk of delayed cerebral ischemia (DCI), hypotension, lung complications, and anemia were elicited during the qualitative interviews using a simple thresholding technique. During the thresholding, the levels of the key attribute were increased to elicit clinicians' maximum acceptable attribute level. The results were used to determine preference-relevant risk ranges to be covered by attributes in the subsequent preference instruments.[1]

The interviews followed a semi-structured interview guide that consisted of three parts:

- **Part 1** included open-ended questions about clinicians' medical background and experience in treating patients with acute subarachnoid hemorrhage (aSAH).
- **Part 2** contained a semi-structured discussion about the risks and benefits of treatments for cerebral vasospasm after an aSAH, and maximum acceptable risks were elicited using thresholding analysis.
- **Part 3** focused on discussing a preliminary question in the choice experiment to understand clinicians' perception of relevant benefits and risks, as well as their willingness to make trade-offs within a hypothetical choice context.

At the end of the interview, all participating clinicians completed a short online survey to collect their sociodemographic and clinical practice characteristics. The interview guide was reviewed by two experienced neurologists with expertise in aSAH and was modified during the data collection phase in response to clinicians' feedback.

All audio recordings of the interviews were transcribed verbatim, and the interview transcripts were analyzed using a combination of content and thematic analysis. Coding of the transcripts involved thoroughly reading through and analyzing each sentence in each transcript. An initial coding dictionary was developed based on the interview notes. The coding dictionary consisted of a list of codes with each representing a topic, opinion, or other data point. The coding dictionary was adjusted after a pilot during which two analysts coded the same transcripts and discussed the outcomes in a workshop to resolve disagreement by discussion. The qualitative analysis of the transcripts took a concept elicitation perspective, which means that the coding dictionaries were developed iteratively with regular workshops involving data analysts and senior researchers of the project. Emerging codes were grouped together into themes and subthemes. Two (20%) of the transcripts were double coded for quality assurance purposes, with disagreements being resolved during a discussion with both analysts.

Ten clinicians completed the interviews, of which five were from the UK (50%) and five were from the US (50%). The clinician sample included two (20%) neurologists (one from the US and UK each), three (30%) neurosurgeons (one from the UK and two from the US), and five (50%) intensivists (three from the UK and two from the US). All clinicians had been practicing

medicine for at least five years and most of them (n=6; 60%) were occupied with their role for approximately 10 to 20 years. Eighty percent (n=8) had treated more than 20 patients with aSAH during the last 10 years.

When asked about the need of a new pharmacological treatment that would reduce the likelihood of clinical deterioration due to DCI following aSAH using a 1–10 range, 80% (n=8) reported a high need with scoring results of either 9 or 10.

The findings from the qualitative analysis were visually summarized in a conceptual map, highlighting the initial management as well as DCI and cerebral vasospasm as themes emerging from the analysis (below).

Conceptual Map

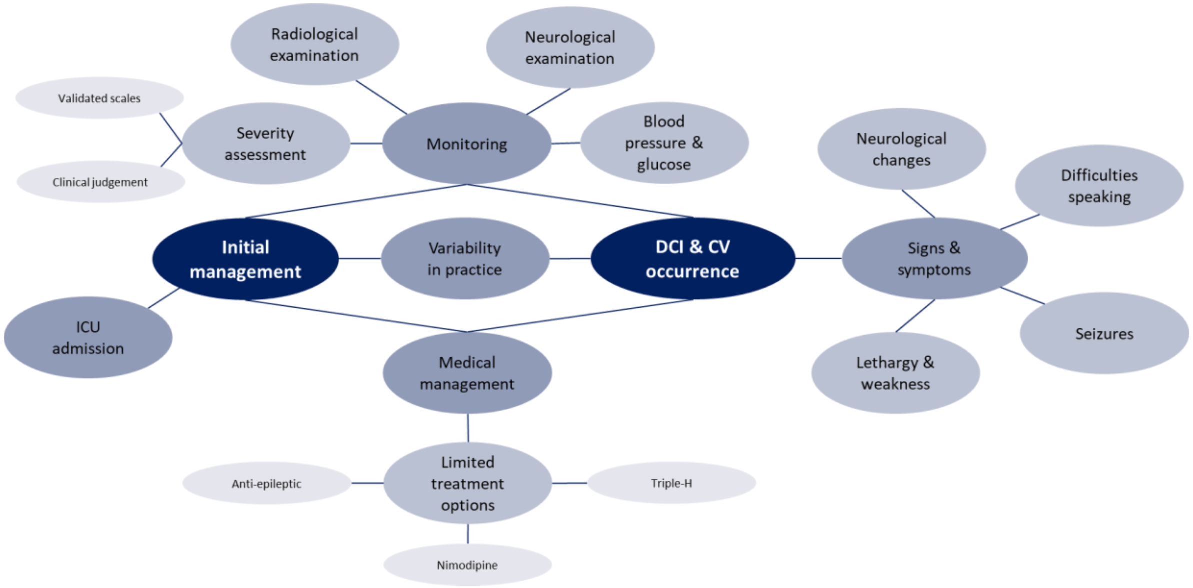

Abbreviations: CV, cerebral vasospasm; DCI, delayed cerebral ischemia; ICU, intensive care unit; Triple-H, induced hypervolemia, hemodilution, and hypertension therapy

Overall, there was a variability in practice in the initial management of aSAH in terms of clinical severity assessment, ICU admission, and medical management after aneurysm repair. However, despite the differences in practice, most clinicians (n=7) reported the use of the Glasgow Coma Scale as an initial measure of consciousness that offers an immediate indication of severity in patients presenting with aSAH. Some clinicians also used other validated scales to determine the initial clinical severity, including the Hunt and Hess (n=6) and Fisher (n=4) scales.[2]

Upon diagnosis of aSAH, some clinicians (n=3) reported guaranteed admission of patients to the ICU or high-dependency unit, while others would only admit patients to the ICU or high-dependency unit depending on their severity and/or after an aneurysm repair (n=6). Medical management post-aneurysm repair varied across clinicians. Some reported the use of anti-epileptics to prevent seizures (n=2) and heparin prophylaxis (n=6), while others emphasized more general monitoring of vital signs, glucose (n=4) and blood pressure (n=5). Across the

interviews, all clinicians reported following some guidelines on the management of aSAH. However, they recognized that there was no consensus on a universally accepted guideline.

Example Quotes on Variability in Management of aSAH

Theme	Participant ID	Quote
Severity assessment	002-001 (Neurosurgeon)	"I think in the first way you use the way that you assess nearly all neurosurgical patients which is that you use the Glasgow Coma Scale … so if someone phones up the hospital in the middle of the night and says I've got someone who's GCS 12 with a subarachnoid hemorrhage, you know that they're not that bad. […] if they phone up and they say we're in A&E, a man's come in with a subarachnoid hemorrhage, he's GCS 7, you know that he's very bad. So, that's the first thing."
	001-102 (Intensivist)	"Fisher Scale that is helpful for determining the presence of subarachnoid hemorrhage on a CAT scan and this seems to have fairly good predictive value for the presence subsequently of vasospasm and diffused cerebral ischemia, so it has a rough correlation with ultimate outcomes and prognosis."
	002-102 (Intensivist)	"There are… we don't tend to… or maybe the radiologists do, but we don't tend to clinically use the other scores like Fisher Scales which you can use to class the… and the CT findings, but yeah, we tend to do it based on the clinical picture and that scale [WFNS]"
ICU admission	001-102 (Intensivist)	"[P]ost-operatively everybody would come to the ICU. But preoperatively, we look for unstable vital signs, we look for risk factors that would increase the chance of a bleed or a rebleed, worsening of the bleed, more cerebral ischemia."

	002-202 (Neurologist)	"[i]t depends on their physiological status basically, I would say probably the majority of people would not necessarily require intensive care admission… they'll certainly have a bed reserved for them subsequent to the treatment of their aneurysm, whether that be clipping or coiling, but that's not necessarily always needed either."
	001-001 (Neurosurgeon)	"Typically, we do, at least for the first few days [post-surgery] and then if they're in good grade meaning that they're neurologically doing well, some will be transferred to the general ward."
Medical management post-aneurysm repair	001-001 (Neurosurgeon)	"[…] because there's about a 15% rate of seizures with aneurysm or in aSAH, I personally routinely give it in everybody - the Keppra, levetiracetam - because it's well tolerated. Typically, I give it for about two weeks."
	002-101 (Intensivist)	"What definitely is required is to basically keep everything normal and avoid you know, too high, too low blood glucose, too low, too high blood pressure, you know, normothermia in most cases. You know, there is a possibility of cooling the patient, but there is no definitive evidence for that either. But basically, to keep everything normal is the guide or the guidance for that"
	001-001 (Neurosurgeon)	"Well you know, a lot of that is institution and surgeon preference. Personally, you know, the rate of seizures and subarachnoid hemorrhage is up to 15%, heparin is a pretty well tolerated drug […]"
Lack of universally accepted guidelines	002-102 (Intensivist)	"There are NICE guidelines available, they're not particularly specific and so I don't find them particularly useful, we've got our own protocols that we follow within the hospital."
	001-002 (Neurosurgeon)	"[T]here is no gold standard that is inviolable, never changing etc., etc."

	002-103 (Intensivist)	"So, we've got our local regional neurosurgical center's guidelines, which is what we follow, which are based on national recommendations."

Abbreviations: A&E, accident and emergency; aSAH, acute subarachnoid hemorrhage; CAT, computerized tomography; CT, computerized tomography; GCS, Glasgow coma scale; ICU, intensive care unit; NICE, National Institute for Health and Care Excellence

Clinicians reported that the most concerning complications following aSAH were re-bleeding, cerebral vasospasm, and DCI. Other complications such as seizure, hydrocephalus and cardiomyopathy were also reported. In terms of the monitoring of the occurrence of cerebral vasospasm and DCI, clinicians reported a combination of neurological and radiological examinations (e.g., transcranial Doppler or computed tomography scan). In addition to the use of the validated scales (e.g., Glasgow Coma Scale, Hunt and Hess scale, Fisher) during neurological examinations, most clinicians (n=6) also indicated the use of their judgment during clinical examinations to recognize changes in the patient's neurological status. All clinicians reported the routine use of nimodipine for the prevention and management of cerebral vasospasm post-aSAH. However, they acknowledged that, at present, there are limited treatment options available, and there is a need for an effective alternative on top of the current standard of care. For a new treatment to be considered effective, clinicians reported that it must be able to reduce the risk of cerebral vasospasm and DCI. On a longer term, some clinicians would like to see improvements in quality of life, such as the ability to conduct activities of daily living upon recovery. The table below summarizes example quotes on the management of cerebral vasospasm and DCI.

Example Quotes on Management of CV and DCI

**Theme**	**Participant ID**	**Quote**
Monitoring of cerebral vasospasm and DCI	001-102 (Intensivist)	"Many times, these people will have a bolt placed in their head to determine intercranial pressure. We use various scales, the GCS for example, although that's really not all that precise, and the neurosurgical scales, the Hunt Hess scale. I mean these sorts of things, but these are coarse measures, and they don't really give us the moment to moment intervention potential that a bolt would."
	002-001 (Neurosurgeon)	"Exactly, so either a global loss, drop in conscious level or a focal or say a seizure or focal signs like a stroke, so difficulty with speech, weakness of an arm or a leg or paralysis of one side of the body or something like that, they're the commonest signs of delayed cerebral ischemia."
	002-101 (Intensivist)	"Well, it depends on the situation, but you know, as you say, a transcranial Doppler would be one, and then the scanning modalities, you know, to assess whether there is vasospasm going on or not. So, it's neurological examination, maybe more subtle tests, non-invasive tests, followed by scans."

Limited treatment options	002-102 (Intensivist)	"But to be honest we were incredibly limited, so really it's only nimodipine that we tend to use in terms of routine….We used to think that statins were a good idea and we used to use pravastatin in big doses but since that's been shown to be not helpful, we don't use that anymore."
	002-101 (Intensivist)	"Not particularly. So, you know, these patients are challenging, they can deteriorate quite quickly, and the challenge is that there are not many treatment options."
	001-102 (Intensivist)	"Okay, well there is a study involving magnesium and I don't think that that is completed with results that are satisfactory. Magnesium is used for a lot of vasoactive diseases, especially preeclampsia… eclampsia for women at risk for eclampsia. Nimodipine is the go-to thing right now. Adenosine and nicardipine and I think there's one other medication that is being researched. I mean, again, I don't think they're ready for prime time yet."
Effectiveness of new treatment	002-102 (Intensivist)	"I think you know, some sort of impact upon reducing cerebral vasospasm, that wouldn't necessarily have to be huge because the devastation, complications of vasospasm on DCI are massive, you can go from being really well to being someone who's going to end up in a nursing home."
	001-102 (Intensivist)	"Well I'd like to see a significant reduction in the incidence of diffused cerebral ischemia and more importantly a better outcome. So, if we look at the… say something like a modified Rankin Scale to determine an improvement in outcome or better functionality in terms of activities of daily living or improvement in speech or improvement in memory."
	002-202 (Neurologist)	"I suppose sort of medium to longer term data that indicates that they improve functional outcome, perhaps rapidity of recovery, reduced mortality compared to best sort of standard care."

Abbreviations: DCI, delayed cerebral ischemia; GCS, Glasgow coma scale; ICU, intensive care unit; NICE, National Institute for Health and Care Excellence

The results from the elicitation of clinicians' minimum acceptable reduction of DCI, hypotension, lung complications and anemia during the qualitative interviews, to inform the selection of adequate levels for each attribute, are presented in .

Results from the Thresholding Analysis During Clinician Interviews

Clinician	DCI	Hypotension	Lung complications	Anemia
001-201	20%	35%	5%	20%
001-101	15%	-	-	100%
002-102	20%	100%	40%	35%
002-101	20%	50%	-	-
001-102	20%	-	-	-
002-202	20%	33%	5%	45%
002-001	20%	<5%	<1%	30%
001-003	10%	<3%	10%	13%
002-103	15%	15%	-	-
001-001	20%	50%	40%	30%

Abbreviation: DCI, delayed cerebral ischemia

Note: Midpoints were used where ranges were reported.

Overall, most clinicians (n=7; 70%) were willing to accept up to a 20% chance of DCI occurrence with the use of an endothelin receptor antagonist (ERA) compared to a 30% chance of DCI occurrence without the use of ERA. Acceptable risk levels varied widely between clinicians, such that further adjustment after the quantitative pilot was expected. Clinicians emphasized the importance of having a clear description of the severity of the AEs to clearly judge the importance of these events.

Finally, all clinicians were presented with a draft choice task. When making choices between two hypothetical alternatives, they considered all the benefit and risk attributes. Clinicians were willing and able to make trade-offs but re-emphasized the need for clearly describing the severity of adverse events. One clinician suggested making the comparison to the ‘no treatment’ alternative clearer. In response to this suggestion, the ‘no treatment’ alternative was moved to the left of the choice task.

Design of the Choice Experiment

The experimental design of a choice experiment describes the combinations of attribute levels that make up the hypothetical profiles from which clinicians choose their preferred treatment. Even a small number of attributes and levels results in far too many possible treatment profiles for clinicians to be presented with. Thus, a subset profiles with desirable mathematical properties was selected to be included in the survey according to an experimental design.[3-5] These profiles were selected such that all effects of interest could be estimated independently and to ensure clinicians need to make trade-offs when choosing.

The full combination of the attributes and levels would have resulted in more than 30,000 choice tasks (i.e., $256 \times (256^{-1}) /2$, where $4^4 = 256$ is the number of possible attribute combinations). A D-efficient experimental design was used to identify the subset of choice tasks that ensures all effects of interest can be estimated independently.[6] The employed D-efficient design minimized the standard errors of the preference estimates for a given sample size. Directional priors were used to minimize the risk of dominant choice tasks. The generated design had 28 choice tasks and was split into two blocks of 14 questions to ensure that the same amount of statistical

information for the different preference parameters would be collected. The design was generated using the software Ngene®. [7]

Within the choice experiment, every choice task asked clinicians to choose the best and the worst treatment out of three unlabeled hypothetical options: 1) ERA A; 2) ERA B; 3) ERA C. This choice format was chosen to maximize the amount of information collected on clinicians' preference structure. Participants were prevented from choosing the same alternative as best and worst. The experimental design systematically varied the attribute levels to ensure clinicians made trade-offs when choosing their preferred alternative. The order of the experimental choice tasks as well as the presentation order of ERA A, ERA B and ERA C was randomized between clinicians. In addition, clinicians were randomized to one of two attribute presentation orders [8]:

- **Order 1** Likelihood of DCI presented <u>before</u> randomly arranged risks
- **Order 2** Likelihood of DCI presented <u>after</u> randomly arranged risks

A practice choice task was used to familiarize clinicians with the format of the choice experiment (Task #0). This was followed by the 14 experimental choice tasks (Tasks #1 to #14).

Qualitative Pre-testing

The survey was qualitatively pre-tested in 60-min interviews with a total of 13 clinicians in the UK (n=6) and US (n=7), over two waves. The first wave included 10 participants, and the second wave included three participants. Interviews were conducted using a 'think aloud' approach, in which the clinicians completed the online survey while sharing their screen, while an experienced interviewer observed their responses and listened to their reasoning. Using a semi-structured interview guide, interviewers also probed clinicians on the clarity of survey instructions, their understanding of the survey content, questions and choice tasks, completeness of response options and the relevance of each attribute included in the preference elicitation tasks. Clinicians were also asked if they perceived the choice tasks to be complete or whether any relevant information or concepts were missing.

The interviews were conducted in two waves, to iteratively test adjustments:

Wave 1 (N=10): Participants in the first wave of interviews completed a standard choice experiment including choice tasks offering two treatment alternatives (ERA A and ERA B) and an opt-out 'No ERA'. Within each choice task, a single-choice option in which participants were asked to choose their preferred treatment was presented to clinicians.

Clinicians were familiar with all attributes and felt that all were at least somewhat important depending on their relative values. Across several interviews, it was observed that for some attributes it was difficult to collect trade-off data, due to their low relative importance (i.e., risks of hypotension and anemia). Further, most clinicians never selected the opt-out (e.g., No ERA), citing the acute nature of aSAH and DCI for this preference, which raised concerns about the opt-out option being dominated and therefore biasing preference estimates. Based on these results, the survey design was updated:

The opt-out was removed, and a third treatment option, 'ERA C', was added to the choice tasks to allow for more trade-off information being collected per choice task.

The choice task design was updated from a single choice design to a best-worst scaling-type 3 design in which participants were asked to select both the best and worst treatments in the tasks. This further allowed for the collection of more trade-off information per choice task.

Levels for both the likelihood of DCI and other treatment-related risks were updated; the number of levels for the likelihood of DCI was updated from 5 to 7, with a 3% difference between levels. Levels were also added to all attributes to incorporate values associated with placebo treatment. This change was used to generate more choice situations in which attributes of lower importance had a meaningful impact on clinicians' choices.

To ensure clinicians traded off between all risk attributes, an overlapped design was employed that forced the likelihood of DCI to take the same value for two alternatives. This again facilitated the collection of trade-off data for attributes of lesser importance.

Minor edits were also made to the descriptive language used in the survey introduction and attribute descriptions to add clarity and improve clinical accuracy.

Wave 2 (N=3): Following updates to the survey design after the first wave of interviews, participants in the second wave completed the revised survey. Clinicians showed a good understanding of the updated instrument and appeared to improve the insights on trade-offs collected in the survey. No further edits were made following completion of the second wave of interviews.

Statistical Analysis

Based on random utility maximization framework, clinicians' choices were modeled as a function of the treatment characteristics (i.e., attribute levels included in the discrete choice experiment) and clinicians' sensitivities to changes in treatment characteristics.

The utility (U) of treatment option (j) for clinician (n) in choice tasks (t) was specified as a fully categorical linear and additive function:

$$\begin{aligned} U_{ntj} = \alpha_A + \alpha_B + \beta_{1n}ANEMIA[10\%]_{ntj} + \beta_{2n}ANEMIA[20\%]_{ntj} + \beta_{3n}ANEMIA[30\%]_{ntj} \\ + \beta_{4n}DCI[12\%]_{ntj} + \beta_{5n}DCI[15\%]_{ntj} + \beta_{6n}DCI[18\%]_{ntj} + \beta_{7n}DCI[21\%]_{ntj} \\ + \beta_{8n}DCI[24\%]_{ntj} + \beta_{9n}DCI[27\%]_{ntj} + \beta_{10n}LUNG[20\%]_{ntj} \\ + \beta_{11n}LUNG[30\%]_{ntj} + \beta_{12n}LUNG[40\%]_{ntj} + \beta_{13n}HYPOTENSION[2\%]_{ntj} \\ + \beta_{14n}HYPOTENSION[8\%]_{ntj} + \beta_{14n}HYPOTENSION[16\%]_{ntj} + \varepsilon_{ntj} \end{aligned}$$

Where ANEMIA corresponds to the risk of anemia, DCI to the likelihood of delayed cerebral ischemia, LUNG to the risk of lung complication, and HYPOTENSION to the risk of hypotension. The α_A and α_B parameters are two constant terms capturing potential ordering effects in clinicians' choices (e.g., systematic tendency to choose option A, everything else being equal). The βs are preference parameters measuring the effect of discrete/categorical changes in the attributes on the probability of preferring the treatment option. For example, β_1 captures the effect of decreasing the risk of anemia from the reference level (i.e., 40%) to 10%. Similarly, β_2 captures the effect of decreasing the risk of anemia from 40% to 20%. The ε component is an error component, which is typically assumed to be identically and independently distributed (as a type I extreme value), leading thus to use of a multinomial logit model for the analysis of clinicians' choices.

The preferences are allowed to vary within the sample by assuming that they are multivariate normally (MVN) distributed.

$$\beta \sim MVN(\mu; \Omega)$$

Where μ is a vector of mean preferences with 14 elements (one for each preference effect), and Ω is the full covariance matrix with 105 elements (14 diagonal and 91 off-diagonal elements) to be estimated.

This model was estimated within the Bayesian framework and is typically referred as hierarchical Bayesian logit model. This model relies on Monte Carlo Markov Chain simulation procedures to estimate the model parameters. The prior distribution for the mean elements was a diffuse MVN distribution with null mean and large variance. The prior distribution for the covariance elements was an inverted Wishart distribution with K degrees of freedom and parameter KI, where I is the identity matrix and K is the number of preference effects.

The Monte Carlo Markov Chain simulation procedure was then used to update these prior distributions with the choice data: 50,000 draws were used before convergence (i.e., burn-in period), 50,000 draws were used after convergence, and every 25th draw was retained, leaving thus 2000 effective draws to simulate the posterior distribution.

References

1. Mørkbak MR, Christensen T, Gyrd-Hansen D. Choke price bias in choice experiments. *Environ Resour Econ.* 2010;45(4):537-551.
2. Mooij JJ. Editorial: grading and decision-making in (aneurysmal) subarachnoid haemorrhage. *Interv Neuroradiol.* 2001;7(4):283-289.
3. Huber J, Zwerina K. The importance of utility balance in efficient choice designs. *J Mark.* 1996;33(3):307-317.
4. Kessels R, Goos P, Vandebroek M. A comparison of criteria to design efficient choice experiments. *J Mark.* 2006;43(3):409-419.
5. Reed Johnson F, Lancsar E, Marshall D, et al. Constructing experimental designs for discrete-choice experiments: report of the ISPOR Conjoint Analysis Experimental Design Good Research Practices Task Force. *Value Health.* 2013;16(1):3-13.
6. Rose JM, Bliemer M. Constructing efficient stated choice experimental designs. *Transport Reviews.* 2009;29(5):587-617.
7. Choice Metrics. Choice Metrics Ngene 1.2 User Manual and Reference Guide. The Cutting Edge in Experimental Design. In:2019.
8. Heidenreich S, Phillips-Beyer A, Flamion B, Ross M, Seo J, Marsh K. Benefit-Risk or Risk-Benefit Trade-Offs? Another Look at Attribute Ordering Effects in a Pilot Choice Experiment. *Patient.* 2021;14(1):65-74.

Survey Instrument

Introduction

Thank you for agreeing to taking part in this online survey. The survey aims to understand the perspectives and treatment preferences of clinicians who are experienced in treating patients with aneurysmal subarachnoid haemorrhage (aSAH).

There are **four** sections in this survey, which will take about 30 minutes to complete:

Section 1 will ask you questions about the treatment characteristics of an endothelin receptor antagonist (ERA).

Section 2 will show you 14 choice questions. Each question will ask you to choose the best and the worst treatment out of three ERA that are available to you. This is an established method that helps us understand what matters to you when you choose a treatment.

Section 3 will explore how specific aspects of ERA treatment effect your choice of treatment.

Section 4 will ask questions about you and your clinical experience in the management of aSAH.

Your responses will be confidential, and your name will never be connected with any of your answers. All data will be fully anonymised and used for medical research purposes.

Thank you for your contribution!

About aSAH and ERA

- Cerebral vasospasm and the resulting delayed cerebral ischemia (DCI) typically occur 4 to 14 days after an aneurysmal subarachnoid haemorrhage (aSAH) and are responsible for high morbidity and mortality in patients. Signs of cerebral vasospasm include loss of consciousness, focal numbness, weakness, paralysis, confusion, dizziness, problems speaking, worsening headache, mood changes, and blurred or double vision.
- Treatment options for cerebral vasospasm and resulting DCI are typically limited to hemodynamic therapy, and rescue therapy. Thus, prevention of cerebral vasospasm is an important objective in the management of aSAH.
- Currently, only the calcium channel blocker, nimodipine, is approved for the prevention of DCI post-aSAH. Recently, a new drug class, the endothelin receptor antagonist (ERA), has been shown to prevent or reverse cerebral vasospasm following an episode of aSAH in clinical trials.
- ERAs are administered as a continuous intravenous infusion. The most common side effects are hypotension, lung complications (primarily due to pleural effusion, pulmonary oedema, and pneumonia) and anemia.

Section 1: About ERA treatment characteristics

In this survey, we will ask you to choose between different hypothetical treatment profiles that you would prefer to use to manage cerebral vasospasm and the resulting DCI following an aSAH.

Each of these treatment is described by four attributes:

1) Likelihood of delayed cerebral ischemia (DCI)

2) Risk of hypotension

3) Risk of anemia

4) Risk of lung complications

When you choose your preferred treatment, it is important to consider all of these characteristics and weigh the pros and cons.

We will now introduce each of the treatment characteristics over the next screens.

WEB PAGE BREAK

The efficacy of an ERA can be measured by its ability to prevent the occurrence of clinical deterioration due to delayed cerebral ischemia (DCI) in patients with aSAH.

The likelihood of a patient developing DCI will be illustrated like this:

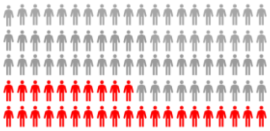

30 out of 100 patients (30%)

The figures in red (30 out of 100 = 30%) represent the number of people who will develop DCI. The figures in grey (70 out of 100 = 70%) represent the number of people who do NOT develop DCI.

The three ERAs (A, B and C) below both reduce the likelihood of DCI occurrence in patients, but to a different degree. Which of the two treatments is more effective, assuming that all other aspects are the same?

	ERA A	ERA B	ERA C
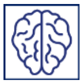 Likelihood of delayed cerebral ischemia	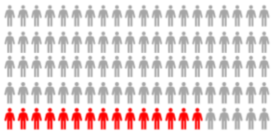 15 out of 100 patients (15%)	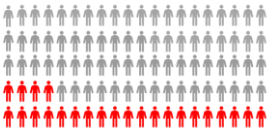 **24 out of 100 patients (24%)**	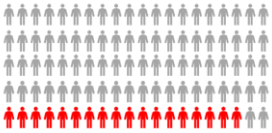 18 out of 100 patients (18%)
	O	O	O

Display instructions

If ERA A is selected: "Correct! With ERA A, only 15 out of 100 patients develops a DCI while ERA B and ERA C carry a higher risk of developing DCI"

If ERA B or ERA C is selected: “NOT correct! With ERA A, only 15 out of 100 patients develops a DCI while ERA B and ERA C carry a higher risk of developing DCI.”

WEB PAGE BREAK

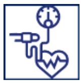

Risk of hypotension

Although ERA have only a small effect on the systemic circulation, some patients may develop hypotension from the use of ERA due to its vasodilation effect. However, the severity of the hypotension is mild to moderate (in the order of 10% reduction in blood pressure) and can be corrected by vasopressor and fluid therapy. Only few patients treated with ERA discontinued treatment due to hypotension.

WEB PAGE BREAK

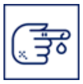

Risk of anemia

Anemia is a class effect of ERA and is attributed to plasma volume expansion as a results of fluid retention. It is typically reversible after discontinuation of ERA and does not require blood transfusion.

WEB PAGE BREAK

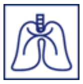

Risk of lung complications

ERAs are associated with lung complications such as pleural effusions, pulmonary oedema and pneumonia. These lung complications are related to fluid retention, which is a known effect of ERAs. Euvolemia can be used for the management of these lung complications within a typical ICU setting.

WEB PAGE BREAK

Please imagine an ERA with the following treatment characteristics:

	ERA
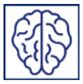 **Likelihood of delayed cerebral ischemia**	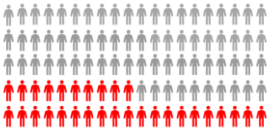 **30 out of 100 patients (30%)**
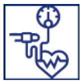 **Risk of hypotension**	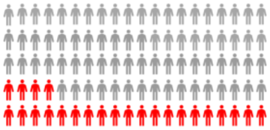 **24 out of 100 patients (24%)**
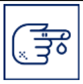 **Risk of anemia**	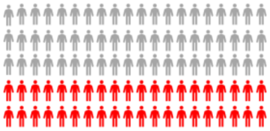 **40 out of 100 patients (40%)**
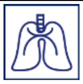 **Risk of lung complications**	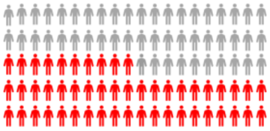 **50 out of 100 patients (50%)**

WEB PAGE BREAK

If you were able to improve one of the four treatment characteristics as described below. Which one of the following treatment characteristics would you choose to improve?

	ERA			Which treatment characteristic would you choose to improve? Please select ONE only.
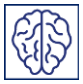 Likelihood of delayed cerebral ischemia	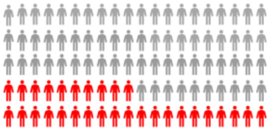 30 out of 100 patients (30%)	→	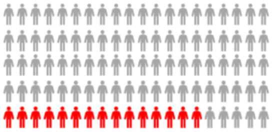 15 out of 100 patients (15%)	☐
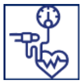 Risk of hypotension	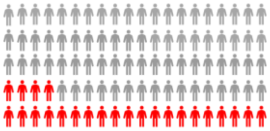 24 out of 100 patients (24%)	→	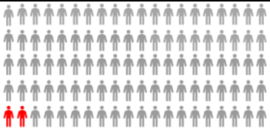 2 out of 100 patients (2%)	☐
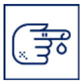 Risk of anemia	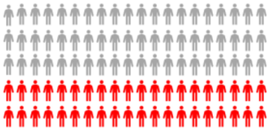 40 out of 100 patients (40%)	→	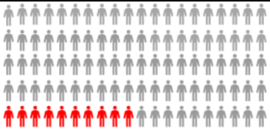 10 out of 100 patients (10%)	☐
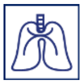 Risk of lung complications	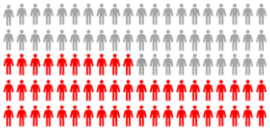 50 out of 100 patients (50%)	→	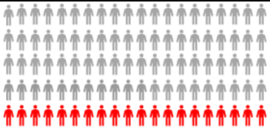 20 out of 100 patients (20%)	☐

WEB PAGE BREAK

Of the remaining treatment characteristics described below, if you could make only one other improvement, which one of the following treatment characteristics would you choose to improve?

NOTE TO PROGRAMMER: REPEAT THE ABOVE QUESTION

FOR EXAMPLE, IF A CLINICIAN CHOOSE TO IMPROVE 'LIKELIHOOD OF DELAYED CEREBRAL ISCHEMIA' IN THIS QUESTION, THEN THE NEXT QUESTION WILL ASK HIM/HER TO CHOOSE FROM 'RISK OF HYPOTENSION', 'RISK OF ANEMIA' AND 'RISK OF LUNG COMPLICATION'.

REPEAT THIS QUESTION 2 TIMES; AT EACH ITERATION, REMOVE THE SELECTED OPTION FROM THE LIST OF COMPARISON.

WEB PAGE BREAK

NOTE TO PROGRAMMER: THE ORDER OF THE CLINICIAN'S CHOICES IN SECTION 1 REPRESENTS THE RANKING OF THE ATTRIBUTES; THE FIRST CHOICE REPRESENTS 1ST RANKED AND SO ON. THIS RANKING INFORMATION OBTAINED IN SECTION 1 WILL BE USED IN SECTION 3 OF THE SURVEY.

Section 2: Understanding your treatment preference

Considering all of the different treatment characteristics between ERA A, ERA B and ERA C, let's take a look at the first practice choice task. When completing the subsequent 14 choice tasks, please imagine the following situation:

> Patient J.J, is a 50-year-old man with a past medical history of hypertension. On arrival in the emergency department, he complained of the "worst headache of his life". He appeared confused but arousable and had no other evidence of neurological deficits. His initial Hunt and Hess (HH) grade was II and his Glasgow Coma Scale (GCS) score was 13. An admission computed tomography (CT) scan of the brain revealed acute subarachnoid haemorrhage due to leakage from a 5-mm anterior artery aneurysm (Fisher grade I). J.J was transferred to the intensive care unit (ICU) where he underwent coiling of his aneurysm. There were no complications during the surgery.
>
> Please imagine this scenario when completing all subsequent choice questions.

Please click <u>here</u> to remind yourself of the patient scenario.

[NOTE TO PROGRAMMER: SCENARIO REMINDER ABOVE ALL CHOICE TASKS]

	ERA A	ERA B	ERA C
Likelihood of delayed cerebral ischemia	30 out of 100 patients (30%)	15 out of 100 patients (15%)	21 out of 100 patients (21%)
Risk of hypotension	2 out of 100 patients (2%)	16 out of 100 patients (16%)	8 out of 100 patients (8%)
Risk of anemia	10 out of 100 patients (10%)	30 out of 100 patients (30%)	20 out of 100 patients (20%)
Risk of lung complications	20 out of 100 patients (20%)	40 out of 100 patients (40%)	50 out of 100 patients (50%)
Which is the <u>best</u> treatment?	☐	☐	☐
Which is the <u>worst</u> treatment?	☐	☐	☐

NOTE TO PROGRAMMER: SUBSEQUENT CHOICES ARE BASED ON THE EXPERIMENTAL DESIGN

Section 3: Understanding how you value risks of treatment effects

NOTE TO PROGRAMMER: IN THIS SECTION, WE WILL PRESENT THREE DIFFERENT PAIRWISE COMPARISONS DEPENDING ON HOW CLINICIANS RANK THE TREATMENT CHARACTERISTICS IN SECTION 1 OF THE SURVEY:

1ST PAIRWISE COMPARISON WILL COMPARE THE 1ST RANKED ATTRIBUTE VS. 2ND RANKED ATTRIBUTE

2ND PAIRWISE COMPARISON WILL COMPARE THE 2ND RANKED ATTRIBUTE VS. 3RD RANKED ATTRIBUTE

3RD PAIRWISE COMPARISON WILL COMPARE THE 3RD RANKED ATTRIBUTE VS. 4TH RANKED ATTRIBUTE

Now that you've told us about your treatment preferences, we would now like to get a deeper understanding on how the different treatment aspects affect your choice of ERA. The following questions are going to help us understand how you value different levels of the treatment aspects.

Please imagine that there are two ERAs that only differ in the following risks:

Please click here to remind yourself of the patient scenario.

	ERA A	ERA B
**[1st ranked attribute]**	**[Worst risk level for 1st ranked attribute]**	**[Vary risk levels for 1st ranked attribute according to experimental design]**
**[2nd ranked attribute]**	**[Best risk level for 2nd ranked attribute]**	**[Worst risk level for 2nd ranked attribute]**
**Which treatment would you choose?**	☐	☐

WEB PAGE BREAK

Now, please imagine that there are two ERAs that only differ in the following risks:

Please click here to remind yourself of the patient scenario.

	ERA A	ERA B
**[2nd ranked attribute]**	**[Worst risk level for 2nd ranked attribute]**	**[Vary risk levels for 2nd ranked attribute according to experimental design]**
**[3rd ranked attribute]**	**[Best risk level for 3rd ranked attribute]**	**[Worst risk level for 3rd ranked attribute]**
**Which treatment would you choose?**	☐	☐

WEB PAGE BREAK

Finally, please imagine that there are two ERAs that only differ in the following risks:

Please click here to remind yourself of the patient scenario.

	ERA A	ERA B
[3rd ranked attribute]	[Worst risk level for 3rd ranked attribute]	[Vary risk levels for 3rd ranked attribute according to experimental design]
[4th ranked attribute]	[Best risk level for 4th ranked attribute]	[Worst risk level for 4th ranked attribute]
Which treatment would you choose?	☐	☐

WEB PAGE BREAK

Section 4: About you and your experience in aSAH management

1. How long have you been practicing medicine?
   Please select only one option

   ☐ Less than 5 years
   ☐ 5-10 years
   ☐ 10-20 years
   ☐ More than 20 years

2. How long have you been practicing in your current role as a [Programmer: insert answer from screening question X]?
   Please select only one option

   ☐ Less than 5 years
   ☐ 5-10 years
   ☐ 10-20 years
   ☐ More than 20 years

3. Several pharmacological treatments have been investigated to reduce the likelihood of clinical deterioration due to delayed cerebral ischemia (DCI) following aSAH by preventing cerebral vasospasm. On a scale of 1 to 10, how do you perceive the need for such a new pharmacological treatment in the routine care of aSAH patients?
   Please choose one option from the scale below

❍	❍	❍	❍	❍	❍	❍	❍	❍	❍	❍
0 Not Needed	1	2	3	4	5	6	7	8	9	10 Very Much Needed

4. *"I think that by avoiding DCI and its associated complications, treatment with an ERA can potentially reduce the ICU stay of patients"*

Please choose an option from the drop-down menu
[DROP DOWN OPTION: not at all; by less than a day; by 1 day; by 2 days; by 3 days; by more than 3 days; Don't know]

5. *"I think that by avoiding DCI and its associated complications, treatment with an ERA can potentially reduce the overall hospital stay of patients"*
Please choose an option from the drop-down menu
[DROP DOWN OPTION: not at all; by less than a day; by 1-2 days; by 2-3 days; by 3-5 days; by 5-6 days; by more than 6 days; Don't know]

6. Which of the following best describe the institution where you are currently practicing?
Please select only one option.

- ☐ University affiliated/teaching
- ☐ Non-university affiliated

7. How many aSAH cases are approximately treated in your institution per year?
Please select only one option.

- ☐ Less than 20
- ☐ 20-40
- ☐ 41-60
- ☐ 61-80
- ☐ 81-100
- ☐ >100

8. When is an aneurysm repair routinely conducted in your institution following an aSAH diagnosis?
Please select only one option.

- ☐ As soon as possible
- ☐ Less than 24 hours following diagnosis
- ☐ Less than 48 hours following diagnosis
- ☐ Less than 72 hours following diagnosis

9. Is there an aSAH management protocol in your institution?
Please select only one option.

- ☐ Yes
- ☐ No

10. How satisfied are you with the current aSAH management protocol in your institution?
Please select only one option.

- ☐ Very dissatisfied
- ☐ Somewhat dissatisfied
- ☐ Neither satisfied nor dissatisfied

- ☐ Somewhat satisfied
- ☐ Very satisfied

11. **Which of the following drugs do you commonly use to prevent cerebral vasospasm following an aSAH?**
    Please select all that applies.

- ☐ Nimodipine
- ☐ Statins
- ☐ Magnesium
- ☐ Nicardipine
- ☐ None of the above

12. **Which of the following method(s) do you commonly employ to diagnosis of cerebral vasospasm following an aSAH?**
    Please select all that applies.

- ☐ Transcranial Doppler
- ☐ CT angiography
- ☐ Conventional angiography
- ☐ CT perfusion
- ☐ Brain tissue oxygen tension
- ☐ None of the above

13. **In the medical management of symptomatic cerebral vasospasm, which of the following technique do you commonly employ?**
    Please select only one option.

- ☐ 'Triple-H' therapy (Hypervolemia–Haemodilution–Hypertension)
- ☐ 'Double-H' therapy (Hypervolemia–Hypertension)
- ☐ Induced hypertension
- ☐ None of the above

14. **In the medical management of symptomatic cerebral vasospasm, what is the blood pressure target that you commonly employ?**
    Please select only one option.

- ☐ MAP >110mmHg
- ☐ MAP >100mmHg
- ☐ MAP >90mmHg
- ☐ MAP >80mmHg
- ☐ No specific target

15. **In the medical management of symptomatic cerebral vasospasm, which of the following vasopressor drugs do you commonly use to increase blood pressure?**

Please select all that applies.

- ☐ Norepinephrine
- ☐ Phenylephrine
- ☐ Dopamine
- ☐ Others: _____________

16. **In the interventional management of symptomatic cerebral vasospasm, which of the following endovascular method do you commonly employ?**

Please select only one option.

- ☐ Angioplasty and intra-arterial vasodilator(s)
- ☐ Intra-arterial vasodilator(s) alone
- ☐ Angioplasty alone
- ☐ None of the above

17. **Which of the following intra-arterial vasodilator drugs do you use to treat symptomatic vasospasm?**

Please select all that applies.

- ☐ Nimodipine
- ☐ Milrinone
- ☐ Papaverine
- ☐ Others: _____________________
